# Supplementary material for: P-TRAP: a Panicle Trait Phenotyping tool
Source: BMC Plant Biol. 2013 Aug 29;13:122. doi: 10.1186/1471-2229-13-122 (PMC3848748; doi:10.1186/1471-2229-13-122)
Supplement: Additional file 7 — P-TRAP source code. The Netbeans project that contains the source code of the application. [file 1471-2229-13-122-S7.zip › javadoc.zip!/javadoc/org-bioalg-widgetfactory/org/bioalg/widgetfactory/ParticleWidget.html]

ParticleWidget (WidgetFacory)


JavaScript is disabled on your browser.


- Package
- Class
- Tree
- Deprecated
- Index
- Help

*org.bioalg.widgetfactory 1.0*

- Prev Class
- Next Class

- Frames
- No Frames

- All Classes

- Summary:
- Nested |
- Field |
- Constr |
- Method

- Detail:
- Field |
- Constr |
- Method


org.bioalg.widgetfactory

## Class ParticleWidget

- java.lang.Object
- - org.netbeans.api.visual.widget.Widget
  - - org.bioalg.widgetfactory.ParticleWidget

- All Implemented Interfaces:
  :   javax.accessibility.Accessible, org.openide.util.Lookup.Provider

  ---

    

  ```
  public class ParticleWidget
  extends org.netbeans.api.visual.widget.Widget
  ```

  This class manages the dealing with particles in the editor.

  Author:
  :   faroq

- - ### Nested Class Summary

    - ### Nested classes/interfaces inherited from class org.netbeans.api.visual.widget.Widget

      `org.netbeans.api.visual.widget.Widget.Dependency`
  - ### Constructor Summary

    Constructors

    Constructor and Description

    `ParticleWidget(java.lang.String labelString,
    java.awt.Polygon polygon,
    java.awt.Color color,
    org.netbeans.api.visual.widget.Scene scene)`

    The constructor function
  - ### Method Summary

    Methods

    | Modifier and Type | Method and Description |
    | --- | --- |
    | `protected java.awt.Rectangle` | `calculateClientArea()` |
    | `java.awt.Color` | `getColor()` |
    | `java.lang.String` | `getLabelString()` |
    | `java.awt.Polygon` | `getPolygon()` |
    | `boolean` | `isfixed()` A particle is fixed if it is changed by the user. |
    | `void` | `setColor(java.awt.Color color)` |
    | `void` | `setIsfixed(boolean isfixed)` |
    | `void` | `setLabelString(java.lang.String labelString)` |
    | `void` | `setPolygon(java.awt.Polygon polygon)` |
    | `void` | `shiftPoints()` Normalize the contour points to the center of the rectangle of the particle. |

    - ### Methods inherited from class org.netbeans.api.visual.widget.Widget

      `addChild, addChild, addChild, addChild, addChildren, addDependency, bringToBack, bringToFront, convertLocalToScene, convertLocalToScene, convertSceneToLocal, convertSceneToLocal, createActions, equals, getAccessibleContext, getActions, getActions, getBackground, getBorder, getBounds, getChildConstraint, getChildren, getClientArea, getCursor, getCursorAt, getDependencies, getFont, getForeground, getGraphics, getLayout, getLocation, getLookup, getMaximumSize, getMinimumSize, getParentWidget, getPreferredBounds, getPreferredLocation, getPreferredSize, getResourceTable, getScene, getState, getToolTipText, hashCode, isCheckClipping, isEnabled, isHitAt, isOpaque, isPreferredBoundsSet, isRepaintRequiredForRevalidating, isValidated, isVisible, notifyAdded, notifyBackgroundChanged, notifyFontChanged, notifyForegroundChanged, notifyRemoved, notifyStateChanged, paint, paintBackground, paintBorder, paintChildren, paintWidget, removeChild, removeChildren, removeChildren, removeDependency, removeFromParent, repaint, resolveBounds, revalidate, revalidate, setAccessibleContext, setBackground, setBackgroundFromResource, setBorder, setBorder, setCheckClipping, setChildConstraint, setCursor, setEnabled, setFont, setFontFromResource, setForeground, setForegroundFromResource, setLayout, setMaximumSize, setMinimumSize, setOpaque, setPreferredBounds, setPreferredLocation, setPreferredSize, setResourceTable, setState, setToolTipText, setVisible, updateResources`
    - ### Methods inherited from class java.lang.Object

      `clone, finalize, getClass, notify, notifyAll, toString, wait, wait, wait`

- - ### Constructor Detail


    - #### ParticleWidget

      ```
      public ParticleWidget(java.lang.String labelString,
                    java.awt.Polygon polygon,
                    java.awt.Color color,
                    org.netbeans.api.visual.widget.Scene scene)
      ```

      The constructor function

      Parameters:
      :   `labelString` - A `String` label.
      :   `polygon` - A `Polygon` that represents the particle contour.
      :   `color` - The contour Color.
      :   `scene` - The `Scene` where this widget will be placed in.
  - ### Method Detail


    - #### isfixed

      ```
      public boolean isfixed()
      ```

      A particle is fixed if it is changed by the user.


    - #### setIsfixed

      ```
      public void setIsfixed(boolean isfixed)
      ```


    - #### calculateClientArea

      ```
      protected java.awt.Rectangle calculateClientArea()
      ```

      **Overrides:**
      :   `calculateClientArea` in class `org.netbeans.api.visual.widget.Widget`


    - #### shiftPoints

      ```
      public void shiftPoints()
      ```

      Normalize the contour points to the center of the rectangle of the particle.


    - #### getColor

      ```
      public java.awt.Color getColor()
      ```


    - #### setColor

      ```
      public void setColor(java.awt.Color color)
      ```


    - #### getLabelString

      ```
      public java.lang.String getLabelString()
      ```


    - #### setLabelString

      ```
      public void setLabelString(java.lang.String labelString)
      ```


    - #### getPolygon

      ```
      public java.awt.Polygon getPolygon()
      ```


    - #### setPolygon

      ```
      public void setPolygon(java.awt.Polygon polygon)
      ```


- Package
- Class
- Tree
- Deprecated
- Index
- Help

*org.bioalg.widgetfactory 1.0*

- Prev Class
- Next Class

- Frames
- No Frames

- All Classes

- Summary:
- Nested |
- Field |
- Constr |
- Method

- Detail:
- Field |
- Constr |
- Method
